# Wrist Trauma: More Than Bones

**DOI:** 10.5334/jbsr.2709

**Published:** 2021-12-31

**Authors:** Maryam Shahabpour, Wiem Abid, Luc Van Overstraeten, Michel De Maeseneer

**Affiliations:** 1 Free University of Brussels, BE

**Keywords:** wrist, fracture, intrinsic ligament, extrinsic ligament, injury

## Abstract

Acute and subacute wrist trauma predominantly consist of fractures of the distal radius in elderly patients and most frequently carpal fractures (scaphoid, followed by triquetrum and hamatum) and avulsion fractures of the ulnar styloid in younger patients, especially in sports-related injuries but also in work activities. The initial radiographs may miss the fractures and result when untreated in complications as nonunion, osteonecrosis, and degenerative osteoarthritis.

Fractures of the distal radius and of the scaphoid may be associated with ligament injuries, most frequently the scapholunate complex, which are often overlooked at the emergency department. Patients without osseous injuries may present intrinsic and extrinsic ligament tears that may lead to carpal instability when they are clinically and/or radiologically missed.

Therefore, in acute and subacute setting, computed tomography may be helpful for the detection of subtle fractures, and magnetic resonance imaging, for the early diagnosis of occult fractures and ligament injuries.

## Manuscript

Wrist trauma may engender serious osseous and soft-tissue injuries. In acute traumatic settings, the difficulty in recognizing fractures of the wrist on the initial radiographs can lead to missed wrist fractures. Unrecognized carpal bone fractures may lead to complications such as nonunion, osteonecrosis, and degenerative osteoarthritis resulting in persistent pain and functional impairment [1,2]. According to the literature, 79% of the carpal fractures are seen in the scaphoid, 14% in the triquetrum, 2% in the trapezium, 2% in the hamatum, 1% in the lunatum, 1% in the pisiform, 1% in the capitatum, and 0.2% in the trapezoid [3,4].

The mechanism of injury is commonly a FOOSH (Fall on an Outstretched Hand) and often dictates the fracture pattern. Clinical signs may be subtle and standard radiographs are often insufficient to detect an obvious fracture; therefore, high clinical suspicion and detailed physical examination are mandatory [5]. Welling et al. found that lunatum and triquetrum (proximal carpal row) fractures are often undetectable on X-rays (0% detected for the lunate and only 20% for triquetral fractures) and trapezoid, capitatum, and hamatum (distal carpal row) fractures are also often occult on X-rays (0% detected for trapezoid and capitatum and only 40% of hamatum fractures). They also demonstrated significant association of hamatum fractures with metacarpal fractures and of distal radius fractures with scaphoid and ulnar fractures. Since 30% of wrist fractures were not prospectively diagnosed on X-rays, a computed tomography (CT) of the wrist could be recommended to perform after negative radiographs when clinically suspected [2].

Schmehl et al. recently described an underreporting of osseous wrist and hand injuries on whole-body computed tomography (WBCT) in polytrauma patients. They found out that a clinical re-examination of polytrauma patients and careful re-evaluation of WBCT with multiplanar reformations are essential to reduce the number of missed osseous injuries of wrist and hand reliably. In symptomatic patients with inconclusive CT results in the post-acute phase after severe injuries, a targeted magnetic resonance imaging (MRI) could be performed to identify occult fractures and soft-tissue injuries [6].

When a fracture is detected, there may be associated injuries of the intrinsic or extrinsic ligaments as well as posttraumatic lesions of the triangular fibrocartilage complex (TFCC). When a fracture is ruled out (by X-rays and when possible, by CT), patients with severe wrist trauma presenting at the emergency department may still have soft tissue injuries, especially acute or subacute ligament and TFCC tears. If severe ligament injuries are undetected and not treated (by immobilization or in some cases by surgical repair), they may lead to carpal instability. The same is true for severe TFCC disruption leading to distal radioulnar joint instability [7].

**Figure 1:**
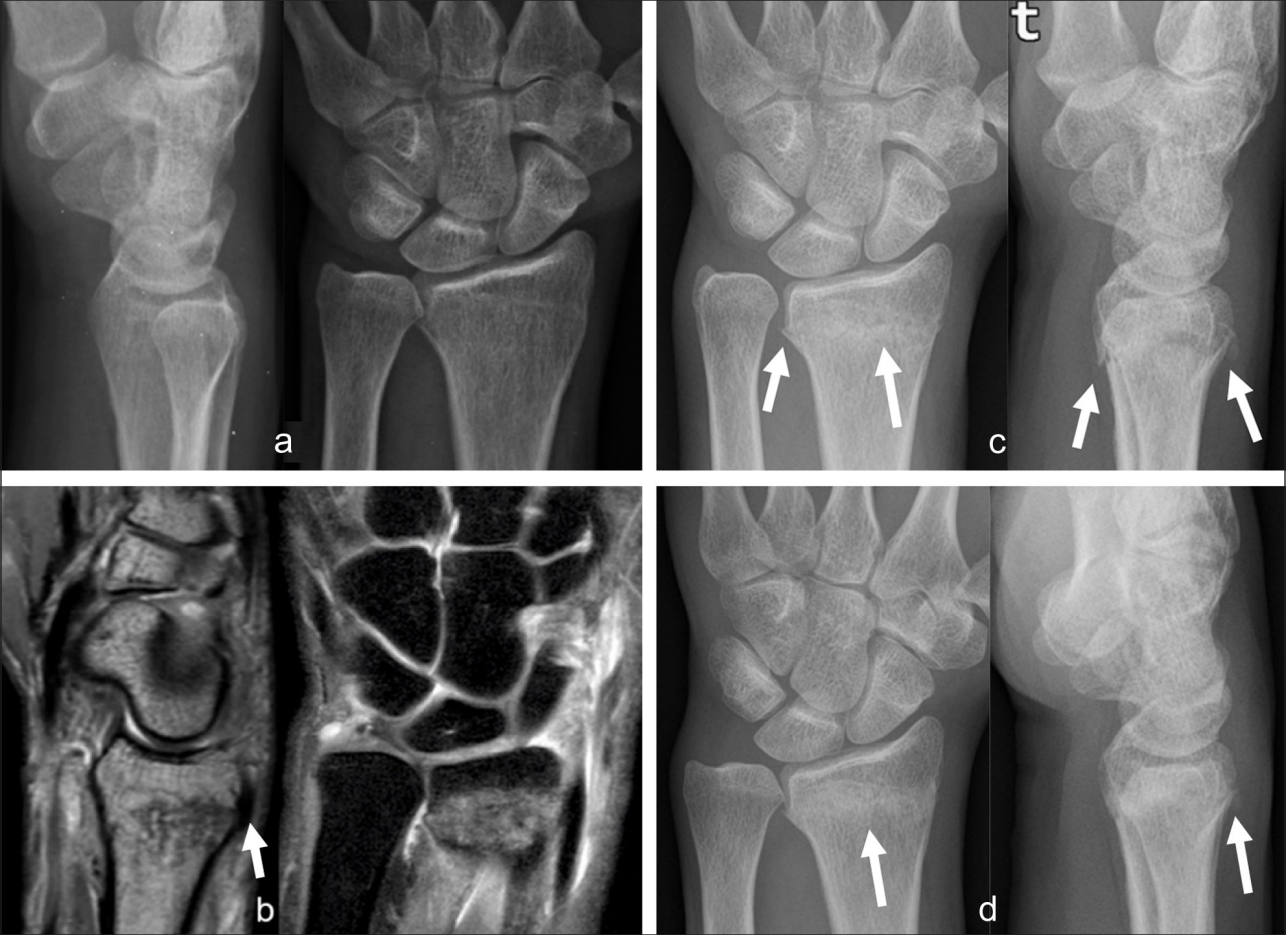
**Occult radius fracture detected by MRI.** The radiographs, (**a**) lateral and anteroposterior views, obtained the day after trauma were negative. Three days later, sagittal PD and coronal PDFS, (**b**) MR images revealed a nondisplaced radius fracture with extensive bone marrow edema and posterior cortical irregularity on the sagittal view (arrow). Control radiographs (AP and lateral views) obtained one month (**c**, arrows) and two months (**d**, arrows) post trauma depict consolidation signs, better seen on d (arrow).

Therefore, based on accurate clinical examination and X-rays, the orthopedic surgeon and the radiologist could, in specific cases, complete the diagnosis by an urgent or semi-urgent MRI of the wrist to detect occult fractures (***Figure 1***) and/or ligament or TFCC injuries.

Apart from complications that will result from undetected fractures as nonunion, scaphoid nonunion advanced collapse (SNAC) and osteonecrosis, the purpose is also not to miss those severe ligament injuries and to prevent development of carpal instability or complications as scapholunate advanced collapse (SLAC). Moreover, in acute and subacute settings (until approximately six weeks after trauma), torn ligaments might still be surgically repaired before extensive infiltration by scar tissue. After this period, surgical treatment is more invasive (arthrodesis) resulting in functional impairment.

Most fractures are encountered at the distal radius and scaphoid. They are often associated with rupture of the scapholunate ligament complex [4,7].

## Radial-Sided Injuries

**Distal radius fractures** (DRF) represent the most common injuries of the upper extremity [7,8,9]. They may be associated to fractures of the ulnar styloid (55%–61%) and distal ulna (6%–9%) and to injuries of the triangular fibrocartilage (39%–43%), scapholunate (SL) ligament (16%–40%), and lunotriquetral ligament (9%–15%) [4,10]. Scaphoid fractures are encountered in 1%–4% of distal radius fractures. Therefore, distal radius fractures should be managed as complex injuries of the wrist [4,11,12].

**Figure 2:**
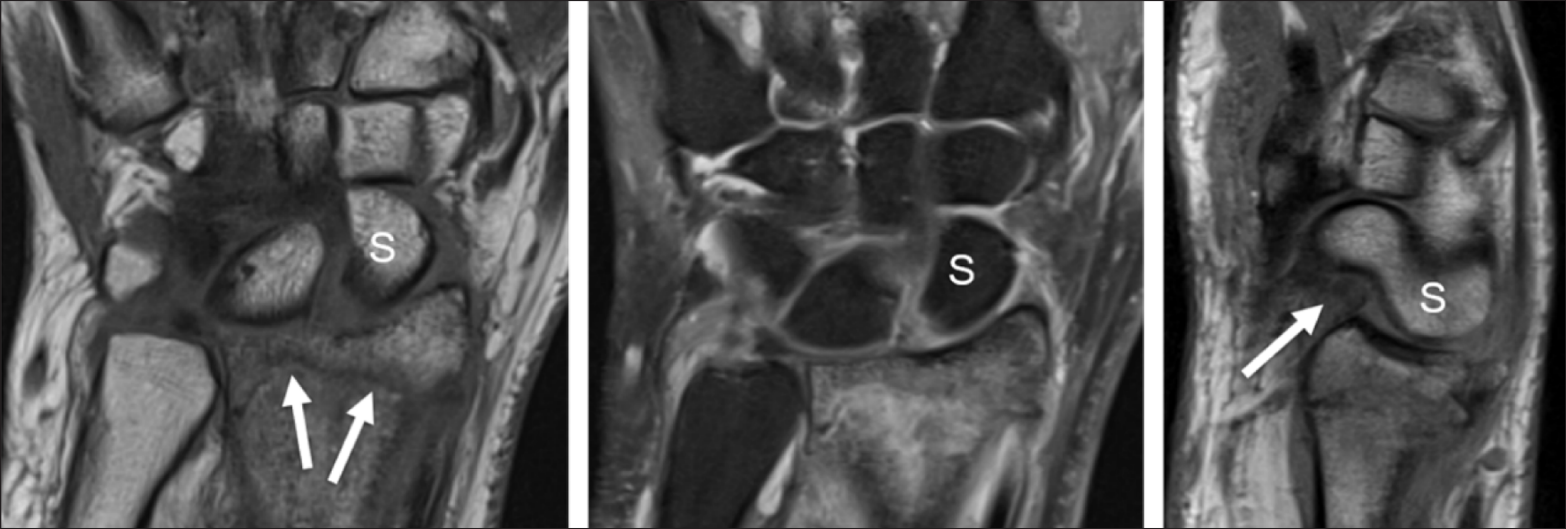
**Distal radius fracture with associated intrinsic scapholunate (SL) and extrinsic palmar radiocarpal ligament rupture (RSC, radioscaphocapitate) detected by MRI.** T1 coronal slice (**a**) shows the fracture line (arrows) and the increased SL space. Proton-density fat saturated coronal slice (**b**) demonstrates high signal surrounding bone marrow edema and increased signal of the SL space. Sagittal proton-density slice (**c**) detects a dorsal avulsion of the distal radius and a torn palmar RSC ligament (arrow) with rotatory subluxation of the scaphoid (S).

In the study of Klempka et al., about 32% of type B and C distal radius fractures (DRFs) are associated with injuries of the SL ligament (***Figure 2***) [7].

No significant correlation is found between the fracture degree and the incidence of SL ligament tears. Three-compartment CT arthrography is convenient because it can simultaneously depict the fractured radius and the injured SL ligament in patients with an intra-articular DRF [7,13,14,15].

**Scaphoid fractures** are the most common injuries to the carpal bones, representing more than 70% of wrist injuries [16]. They are outstanding considering the potential complications in case of delayed diagnosis and treatment (nonunion, delayed union, SNAC or avascular necrosis) [17].

The diagnosis is often difficult and missed at the emergency department. A non-displaced scaphoid fracture may not be visible on standard or even dynamic radiographs. Ultrasound may show hemarthrosis or cortical bone interruption. Plain CT may detect an osseous fissure. However, MRI is the preferred examination to confirm a suspected fissure and differentiate it from a bone contusion. Unfortunately, MRI is still difficult to obtain in the acute stage, and the fissure is only diagnosed when the lesion is healed. In these conditions, arthroscopy can be helpful, as it allows diagnosis and therapeutic control. In athletes, particularly high level athletes, non-displaced stable fractures are stabilized with screwing as the first choice to limit disability to two or three weeks. However, the rate of complications (as nonunion, osteoarthritic degeneration, material breakdown) associated with demanding sports is significant. The therapeutic alternative for non-displaced fractures (considered by most authors to be the treatment of choice) is a long-term brachiopalmar immobilization for up to 10 weeks. When the fractures are displaced, the universally recognized therapeutic indication is an arthroscopic reduction (to limit devascularization related to surgical approach) and a stabilization by cannulated screw, guided on a pin [18].

The descriptive classification reported by Russe details horizontally oblique fractures and transverse or vertically oblique fractures [16,19]. In the AO classification, scaphoid fractures are subdivided into type C1 (ligament avulsion), type C2 (potentially unstable: horizontal, transverse and oblique fractures), and type C3 (unstable: vertical or multiple fragment fractures). Based on original reports describing fracture location, Ten Berg et al. state that waist fractures occur in 66%–82% of the cases [20,21]. Considering fracture plane orientation, transverse fractures are the most frequent (36%–60%), followed by horizontal oblique fractures (30%–47%). Considering fracture stability, most fractures are described as stable (53% and 71%) [19,21].

**Figure 3:**
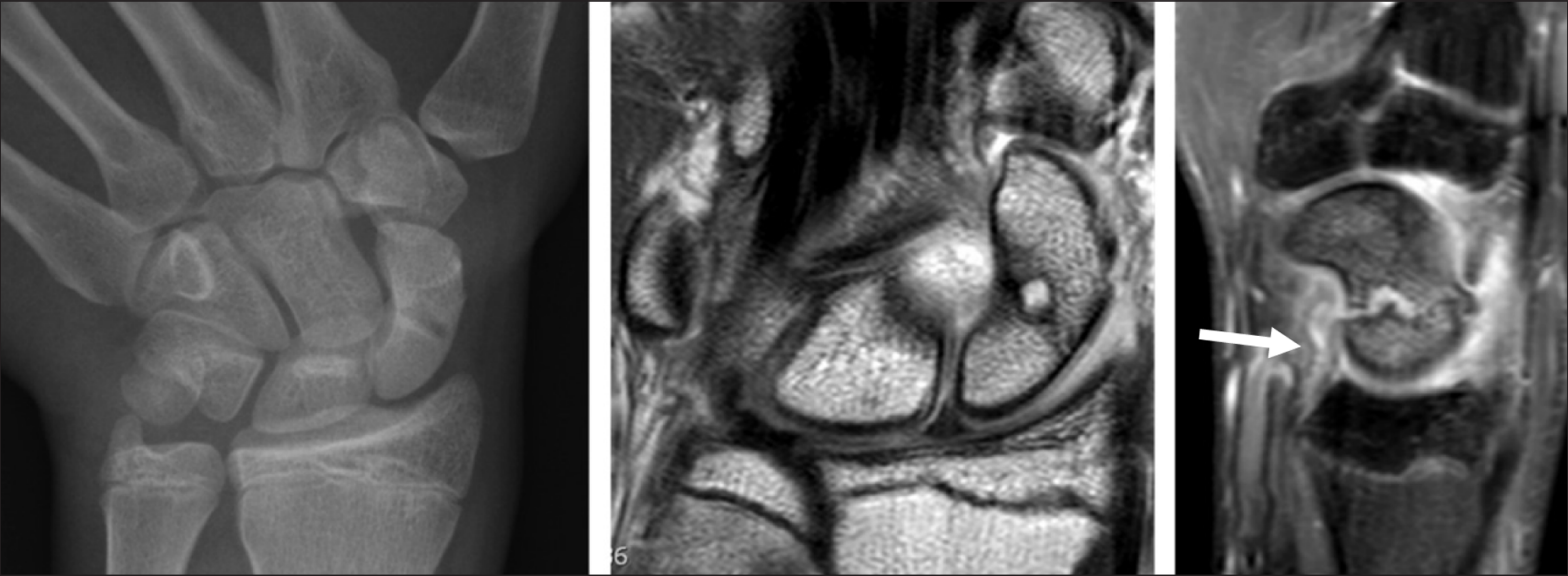
**Evolution of scaphoid fracture with pseudarthrosis in a 17-year-old male.** Radiograph obtained in AP view with ulnar deviation (**a**) one month after trauma discloses an evident fracture of the scaphoid middle third (which was not visible on the first radiographs performed on the day of trauma). As the wrist remaining painful and bone scintigraphy performed one year after trauma was positive at the level of the scaphoid, MRI was obtained to exclude an avascular necrosis at the proximal pole of the scaphoid. Coronal proton-density slice (**b**) and sagittal fat saturated proton-density slice (**c**) revealed pseudarthrosis without osteonecrosis and an extensive bone marrow edema of the whole scaphoid with surrounding soft tissue edema and elongation of the palmar RSC ligament (arrow) on c.

**Complications of scaphoid fractures:** A frequent complication of scaphoid fracture is the lack of consolidation, leading to therapeutic difficulties, especially when it concerns a proximal pole fracture which can critically interrupt the intraosseous vascular network (***Figure 3***).

Plain CT confirms the discontinuity between both scaphoid fragments. MRI can analyze the vascularization of the fragments. Arthroscopy helps to control the reduction, the position of the grafts and the stability of the assemblage and avoids aggravation of the fragments’ devascularization. Grafting from the Lister tubercle could be performed with immediate placement within the area of bone loss. Intrascaphoid and scapholunate wiring is preferred to screwing, especially for very proximal, stage I or II Schernberg fractures, because screwing destroys a larger bone capital. If not treated, hypoxia of the proximal pole can lead to pseudarthrosis and osteonecrosis. Scaphoid nonunion with osteonecrosis may result from overlooked fractures, insufficient immobilization and severe displacement of bone fragments [16,18,20,22,23].

**Figure 4:**
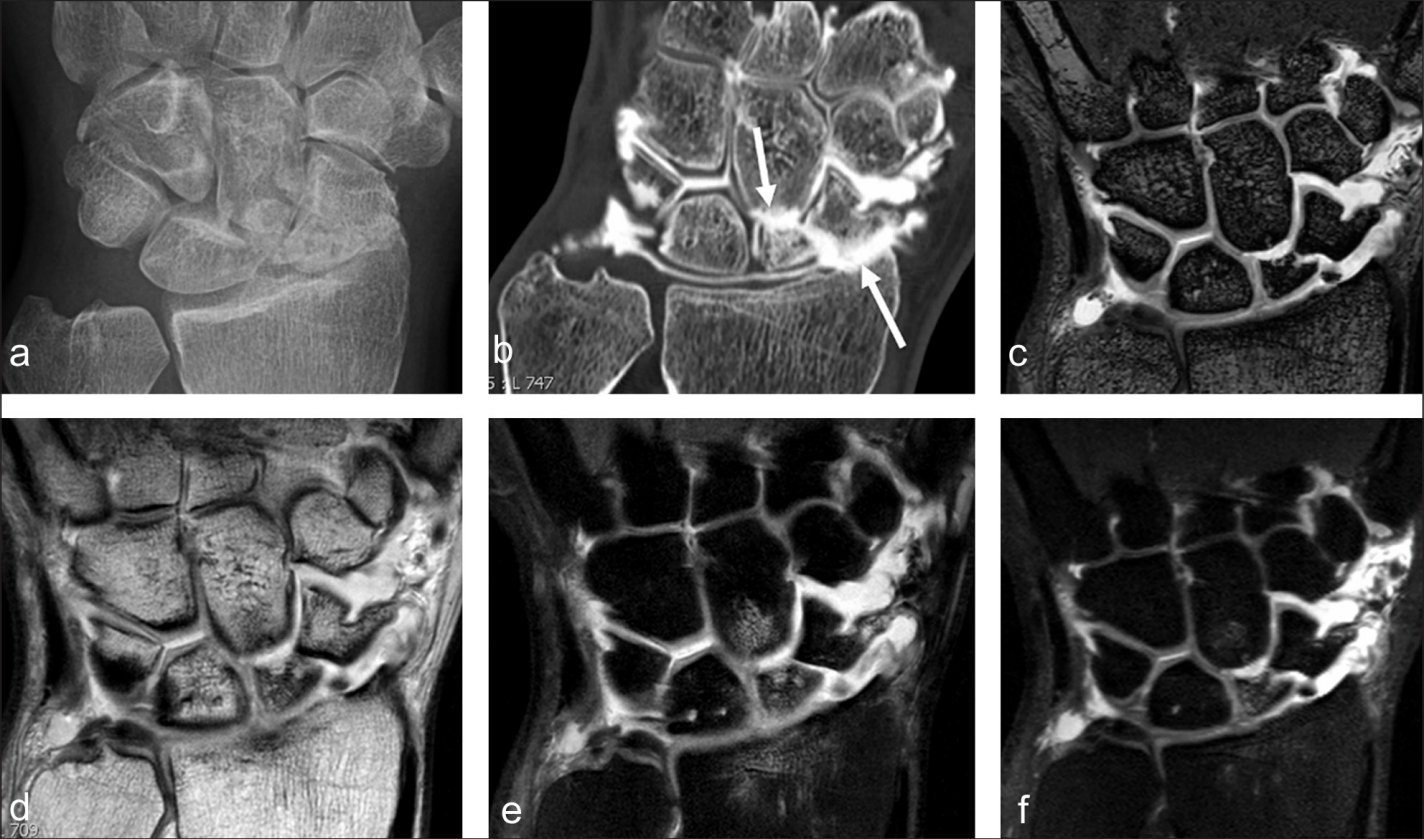
**SNAC 2 wrist after scaphoid fracture with nonunion in a 41-year-old man.** Secondary cartilage degeneration is first seen in the radioscaphoid compartment (**b**, arrow pointing up) and later in the scaphocapitate midcarpal joint space (**b**, arrow pointing down) on conventional radiograph and much better on CT-arthrography (**b**). MRI well demonstrates the nonunion (on 3D DESS with 0.4mm sections) and increased signal in the proximal scaphoid pole on 2D (**e**) and 3D (**f**) proton-density fat-saturated images, corresponding to edema and indicating that the separated fragment might be unstable (and painful). Subchondral bone marrow edema is also detected in the proximal pole of the capitate under the cartilage loss and in the distal radius (**e**).

**Pseudarthosis** is the most serious complication of an untreated scaphoid fracture, resulting in periscaphoid osteoarthritis, followed by carpal structural collapse and radio- and midcarpal osteoarthritis, so-called **Scaphoid Nonunion Advanced Collapse** or **SNAC wrist** (***Figure 4***).

A SNAC wrist starts with a focal arthrosis on the radial styloid process (SNAC 1), arthrosis of the entire radioscaphoid compartment (SNAC 2), and a transition to midcarpal arthritis (SNAC 3). Arthrosis is graded on radiographs of the entire wrist [16]. Crema et al. demonstrated that the distribution of cartilage damage following scaphoid nonunion does not necessarily follow the pattern of osteoarthritis proposed by the staging systems available for SNAC [24]. In patients with scaphoid nonunion assessed on MDCT arthrography, the radial styloid-scaphoid, the STT, the scapho-capitatum, and the proximal radio-scaphoid joints, in addition to the dorsal aspect of the joints, are most commonly affected by degenerative cartilage damage. STT involvement indicates midcarpal involvement that can be the source of midcarpal pain; it could affect management of SNAC [24,25]. Although invasive, CT arthrography is able to exhibit the full spectrum of degenerative cartilage damage in patients with scaphoid nonunion, which is not always true for radiographic assessment. CT arthrography can directly assess cartilage morphology and provides high spatial resolution images with multiplanar assessment [24,26]. Proximal scaphoid nonunion is the most associated fracture location with a global cartilage damage [24]. During this evolution that could last several years, the wrist can remain asymptomatic for a very long time, even under regular increased mechanical stress. Only symptomatic wrists will need surgical treatment. There is indeed no reason to offer to a painless wrist a treatment that could reduce functional performance (mobility or grip strength) without guarantee of definitive stabilization [18].

In association with scaphoid injury, contusions and tears of the SL ligament could be seen, following the same mechanism of injury as falling on an outstretched hand [17]. When a rupture of the carpal ligament stabilizers is left untreated, it leads to carpal collapse, first reducible and becoming later irreducible with associated cartilage damage. **Scapholunate advanced collapse** or **SLAC wrist** is a progressive form of wrist osteoarthritis, frequently encountered after a non-treated traumatic injury of the SL ligament [27,28]. It is due to SL instability (disruption of the scapholunate ligament, which causes SL dissociation and diastasis, rotary subluxation of the scaphoid and DISI). Three stages of SLAC arthropathy are described: cartilage lesions also start on the radial aspect of the radioscaphoid joint compartment (SLAC I). At this stage, radial styloidectomy using arthroscopic approach (to respect the still intact stabilizers) can provide temporary relief (sometimes on a long term). When the articular lesions involve the entire scaphoid fossa (SLAC II), interposition with hanging of a palmaris longus tendon graft can complete the styloid resection. Later cartilage lesions involve the capitolunate joint (SLAC III) with sparing of the radiolunate joint until very late in the osteoarthritic disease process. More pronounced separation between the proximal pole of the scaphoid and the lunate is seen in the late stage. The capitate migrates proximally, displacing the lunate ulnarward, leading to the SLAC pattern of osteoarthropathy [28]. Lunocapitate osteoarthritis can be treated, when necessary, by resection of the proximal pole of the capitate and tendinous interposition. The alternative for both procedures is the wrist denervation, which can also provide a temporary improvement and prolong a career for a few seasons in case of severe pain with preserved mobility. However, after 10 years on average, pain can reappear [18].

To prevent those complications, early detection of occult scaphoid fractures and SL injuries is needed. For the diagnosis of radiographically **occult scaphoid fractures**, MRI remains the gold standard [17,29,30]. Bone marrow edema is detected on fat saturated T2-weighted images and the absence of marrow edema excludes a recent fracture. To improve diagnostic accuracy, a T1- weighted sequence can be added to visualize the fracture line [17].

The fracture of the proximal pole of the **capitate** represents 1% of carpal fractures. It has a bad prognosis in transscaphoid perilunate fracture (Fenton’s syndrome). This requires reduction under open surgical approach and stabilization. The fragment remnant is rarely viable without arthritic degeneration [18].

The stability of the wrist is mainly ensured by the integrity of the carpal bones and joint surfaces and by intact intra-articular (particularly the scapholunate interosseous ligament) and intracapsular ligaments. The severity of joint instability depends on the extent of the underlying ligamentous and/or osseous injury [31,32]. **Injuries to the carpal ligaments** often lead either to functional impairment of the wrist (dynamic instability) or to malalignment of the carpal bones (static instability). Although treatment of these conditions may be difficult, early diagnosis is imperative for a good result. The main goal of the exploration of the ligaments in acute or subacute stage is to recognize injuries that could progress unfavorably and would require a specific treatment [33]. Sprains, mid-substance tears, avulsions, and subacute or chronic stage fibrous infiltration of carpal ligaments could be identified on MR images using 3D fat-saturated PD and 3D DESS (Dual Echo with Steady-State Precession) sequences with maximum 0.5 mm-thick slices [34,35].

Traumatic lesions of the **SL ligament complex** are the most frequent wrist ligament injuries and the most common cause of carpal instability [35,36]. The SL ligament complex comprises not only the intrinsic SL ligament (with dorsal and palmar bands) but also the extrinsic palmar radiocarpal (RC) ligaments: radioscaphocapitate (RSC), long radiolunate (LRL), short radiolunate (SRL), and the intrinsic midcarpal scaphotrapeziotrapezoid (STT) ligament complex at the palmar side. It also includes the extrinsic dorsal radiocarpal (DRC) ligament, the dorsal intercarpal (DIC) ligament, and the dorsal capsular scapholunate septum (DCSS) at the dorsal side [36,37,38] (***Figure 2***).

The scaphoid attachment of the dorsal SL band is weaker and more likely to avulse than the stronger lunate attachment [39,40,41]. The DIC ligament is frequently injured during a fall on an outstretched hand, resulting in a so-called dorsal wrist sprain [42]. Özkan et al. found that patients with MRI findings of a SL ligament tear should also benefit from MRI to assess the integrity of the DIC and DRC ligaments for surgical decision making. If the DIC is structurally compromised, a tenodesis procedure may be preferable [43].

The extrinsic palmar RC ligaments are the most important stabilizers of wrist motion [44,45,46,47]. Taneja et al. studied the prevalence of extrinsic ligament injuries on MRI and its association with intrinsic ligament tears; they found out that the most frequently injured extrinsic ligaments are the palmar LRL and RSC, often injured concomitantly, and the dorsal radiotriquetral (DRT) [36,48,49]. If the RSC ligament is injured together with a rupture of the intrinsic SL ligament, this may lead to scaphoid instability with DISI deformity and SL dissociation with a SL gap on radiographs [50]. Severe injury to the LRL ligament (that normally prevents ulnar translation of the carpal bones) can destabilize the perilunate region and may lead to perilunate dislocation of the carpal bones [36,51].

## Ulnar-Sided Injuries

The most common injuries to the distal ulna are the **ulnar styloid fractures**, usually combined with distal radius fractures and Galeazzi’s fracture of the mid to lower diaphysis of the distal radius with dislocation of the distal radioulnar joint (DRUJ). Posttraumatic instability of the DRUJ with subluxation generally follows injuries of the triangular fibrocartilage complex (TFCC) or of the ulnar styloid to which the TFC attaches [52]. The **TFCC** is frequently affected by fractures of the distal radius [53]. MRI and better with intraarticular contrast injection (MRA) is the imaging modality of choice (both in sensitivity and specificity) to document lesions of the TFCC, whether peripheral or foveal [54]. It can precisely localize the tear and detect associated lesions as a ganglion cyst or a tendinopathy of the carpal extensor tendons. However, MRA cannot determine the degree of TFCC instability, its healing potential or the precise state of DRUJ cartilage. These three criteria are essential in the therapeutic choice between repair and graft reconstruction and can be established by arthroscopy [55]. In recent years, the arthroscopic semiology of TFCC tears has become more precise and a new classification of acute TFCC lesions, specifying Palmer type 1B rupture, permits to provide a therapeutic algorithm [55,63]. Atzei differentiates ‘*acute distal peripheral*’ lesions (isolated, without instability of the DRUJ) which are sutured, from ‘acute proximal foveal’ lesions (detected by the arthroscopic “Hook test”), which are attached to the distal ulna by anchoring or through a transosseous tunnel, ‘*acute combined proximal-distal*’ lesions (almost always responsible for DRUJ instability, except when the oblique distal band is intact), and ‘*multiple acute irreparable*’ lesions [55]. Peripheral arthroscopic reattachment can be achieved by direct foveal approach or through the 6th compartment especially in case of associated lesion of the extensor carpi ulnaris (ECU) tendon or tendon sheath. MRI will therefore contribute to the orientation of the arthroscopic approach. Foveal re-fixation also allows to tighten elongated ulnocarpal ligaments. When the TFCC is irreparable and the cartilage is preserved, reconstruction can be performed by ligament graft. Arthroscopic assistance limits the surgical approach and post-operative stiffness [18].

**Figure 5:**
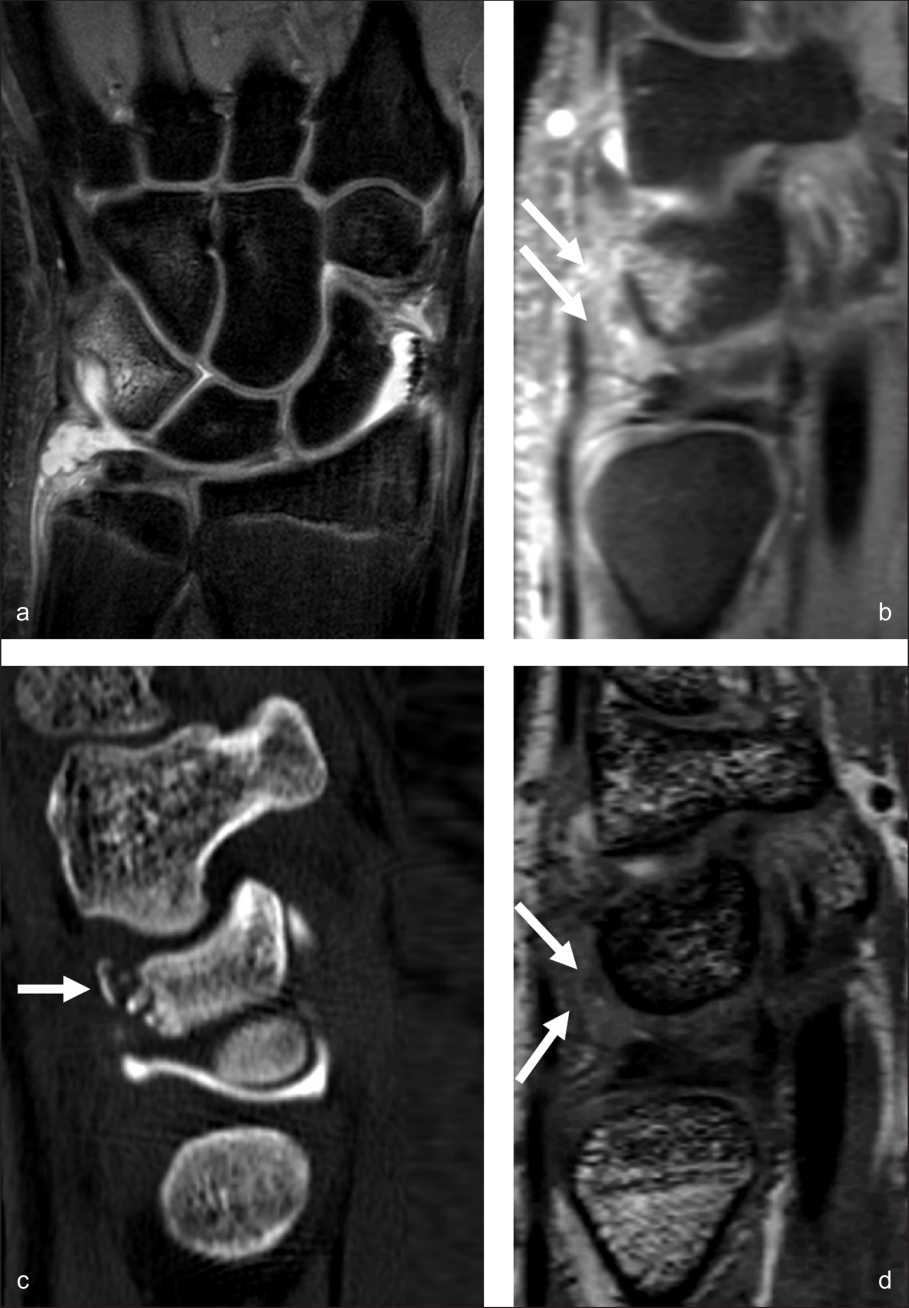
**Triquetrum occult fracture with synovitis in the prestyloid recess and traumatic bone avulsion with subacute edematous infiltration of the dorsal capsular ligaments.** MR arthrography performed 12 days after a fall on an outstrechted hand allows detection of bone marrow edema in the dorsal part of the triquetrum on coronal proton-density fat-saturated images (**a**) and sagittal 0.4mm reconstruction from a 3D proton-density fat-saturated sequence (**b**), as well as edematous infiltration of the surrounding dorsal soft tissues (arrows on **b**). The dorsally avulsed bony fragments are better seen on CT (arrow on **c**). The dorsal capsular infiltration (mostly the extrinsic dorsal radiotriquetral ligament) with low to intermediate signal intensity is also depicted on the sagittal reconstruction from the 3D DESS sequence (arrows on **d**).

**Triquetrum fractures** account for 13% of carpal fractures and have a favorable prognosis and are almost never associated with carpal instability. The treatment is conservative (orthopedic). The mechanisms of injury in triquetrum fractures could be a dorsal bone avulsion by the extrinsic dorsal radiocarpal (DRC) ligament or the dorsal band of the lunotriquetral intrinsic ligament, impaction by the ulnar styloid or shearing by the proximal pole of the hamate. Avulsions of the dorsal rim are more frequent, occurring after a fall with the wrist in extreme flexion [5,52,56,57]. Becce et al. reported that dorsal fractures of the triquetrum are frequently associated with DRT ligament injuries [58]. Traumatic bony avulsion fractures of the dorsal triquetrum may also be associated with edematous infiltration and thickened appearance of the DIC ligament attachment with abnormal signal suggestive of a partial tear. When it is injured, the extrinsic dorsal radiotriquetral (DRT) ligament is as destabilizing for the carpal bones as when the palmar RSC and scaphotrapezial ligaments are injured. In more than 50% of cases of carpal instability, the DRT ligament is injured, usually in association with intrinsic SL ligament damage [36,38,44,59] (***Figure 5***).

**Lunatum fracture** is rarely posttraumatic; in that case it is usually associated with perilunate carpal dislocation. Associated instability can then be related to the direction of the fracture line: radiocarpal instability for transverse fractures and midcarpal instability for palmar fractures. Lunatum fracture often corresponds to a beginning collapse associated to avascular necrosis [18,23].

The **lunotriquetral (LT) compartment** is mainly supported by the intrinsic **LT ligament** and secondary stabilizers, mainly the extrinsic palmar LRL, UT, and DRT ligaments. These secondary stabilizers can temporarily compensate the function of the ruptured SL or LT in stages I and II scapholunate and lunotriquetral instability [36,38,60,61,62]. The extrinsic palmar ulnocarpal ligaments include the ulnotriquetral (**UT**), ulnolunate (**UL**) and ulnocapitate (**UC**) ligaments. At the dorsal side are the extrinsic dorsal ulnotriquetral (**dUT**) ligament and the triquetral attachment of the extrinsic DRC ligament and of the midcarpal DIC ligament. The ulnocarpal ligaments originate perpendicularly on the palmar radioulnar ligament (or the palmar rim of the TFCC) [36].

The extrinsic **ulnocarpal ligaments** may avulse in severe traumatic injuries to the TFCC. Traumatic avulsions of the distal attachments of the TFCC are not common. They are graded as type IC lesions according to Palmer’s arthroscopic classification [63]. Lesions can be identified on thin 3D or 2-mm-thick proton-density (PD) and PD fat-saturated coronal images. The dUT ligament originates on the ulnar styloid and inserts on the dorsal aspect of the triquetrum with a wide attachment that it shares with the DRT ligament. In 2011, Theumann et al. described a tear of the dUT ligament and a bucket-handle tear of the distal lamina of the TFCC attachment on the ulnar styloid (with separation of the distal radioulnar ligaments from the articular disc) in the same patient [64]. Becce et al. found that 81% of dorsal triquetrum fractures were associated with dUT tears in the injured wrists and that dUT tears may be correlated to specific fracture patterns as a proximal tilt of the avulsed triquetral bony fragment [58].

The intrinsic LT ligament injuries, which are less common than SL tears, can also lead to dynamic and static instability. Isolated rupture of the LT ligament or combined injury of LT ligament and one of the main secondary stabilizers of the LT compartment (palmar LRL, palmar UT, DRT) are quite rare. An isolated LT tear is not sufficient to cause a VISI deformity. The adjacent extrinsic ligaments (LRL, pUT, and DRT) must also be injured to produce complete lunotriquetral instability [36].

LT ligament injury may occur with a fall on an outstretched hand in maximal extension, radial deviation, and internal pronation [36,41]. Complete or partial ligamentous disruptions can be visible on MR images; lack of lunotriquetral joint widening makes the diagnosis difficult. Palmar and dorsal LT tears are nearly always associated with proximal segment tears, as in the SL ligament tears [65]. The triquetral attachment of the palmar LT band is weaker and more likely to avulse than the stronger lunate attachment [36,39,40,41]. Association of LT dissociation with degenerative Palmer IID and IIE TFCC lesions is also frequent [36,47,60,63].

**Figure 6:**
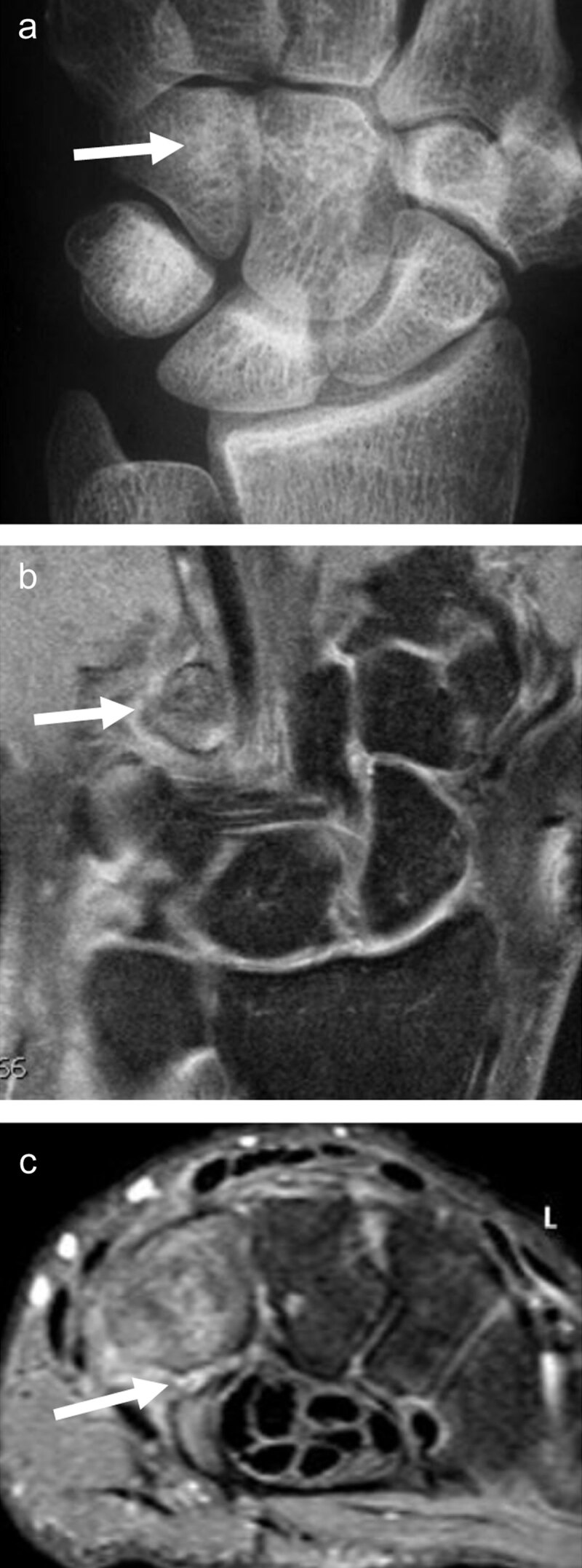
**Fracture of the hook of the hamatum, easily missed on conventional radiographs and on fat-saturated MR images.** The AP X-ray (**a**, arrow) demonstrates the absence of the ring sign (normally present at the level of the hamulus) on the hamate bone. On palmar proton-density fat-saturated coronal image (**b**, arrow), the presence of BME within the hamulus may be obscured because of the close signal intensity of the surrounding muscles. The fracture line at the base of the hamulus could also be missed on the axial proton-density fat-saturated sequence (**c**, arrow).

**Hamatum fractures** represent approximatively 1.7% of all carpal fractures and the hamulus (hook of the hamate) can be injured depending on the mechanism of injury [66]. **Hook** fractures are frequent in racket sport athletes, baseball players, and golfers (***Figure 6***).

**Figure 7:**
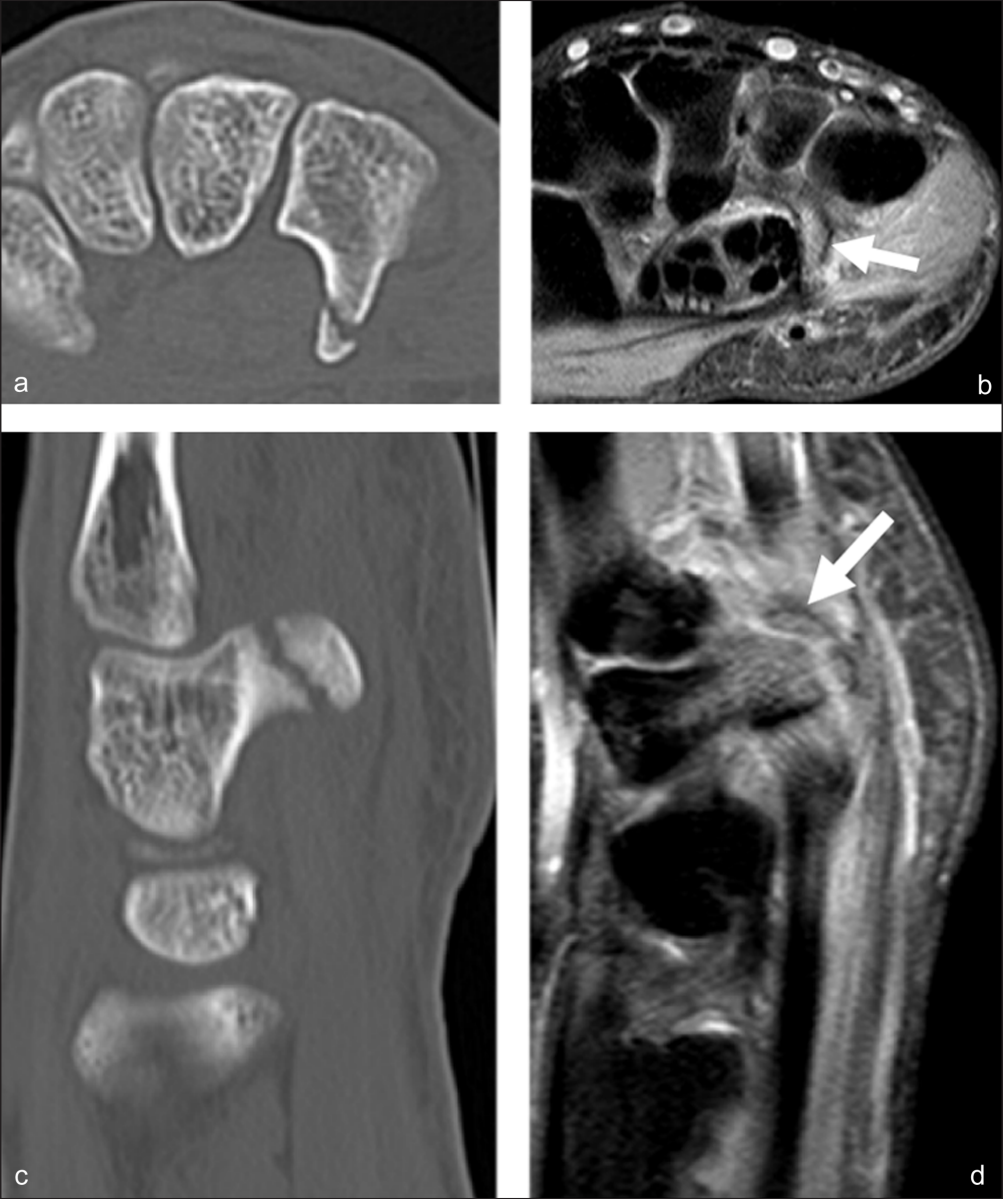
**Another case of direct compression of the hook of the hamate.** Axial CT (**a**) and sagittal reconstruction (**c**) disclose the avulsion fracture, much easier than the corresponding axial (**b**, arrow) and sagittal (**d**, arrow) proton-density fat-saturated MR images.

Direct compression of the hook of hamate represents the most common mechanism of injury, although other mechanisms include **avulsion fractures** of the pisohamate ligament [5,67] (***Figure 7***).

Mandegaran et al. state that the hook of hamate can also be injured by indirect means via a fall on the outstretched hand causing avulsion of the hook by its ligamentous attachments, as the wrist is forced into dorsiflexion in association with a scaphoid fracture [68,69]. An occult fracture of the hook of the hamate is present in 10% of scaphoid fractures. Given the potential morbidity and therapeutic outcome associated with hook of hamate fracture, an assessment of this area is important when investigating scaphoid injury. CT images easily detect the area of cortical discontinuity. However, the fractures are often minimally displaced and could be overlooked on CT. MRI shows bone marrow edema; however, it is easily missed in this small anatomic area, especially on coronal fat-saturated sequences (***Figure 6***). In general MRI is the most reliable imaging method for identifying minimally/non-displaced fractures such as the scaphoid or hook of hamate and the modality of choice for detection of any radiographically occult concomitant fractures in patients with scaphoid fracture [30,68].

The fracture of the hamulus of the hamate is easily missed and hook fractures are frequently complicated by nonunion. Hamulus nonunion is rarely asymptomatic because this part of the hamate corresponds to the distal ulnar attachment of the transverse carpal ligament (or flexor retinaculum) [68,70]. Chronic symptomatic nonunion (including chronic ulnar-sided wrist pain, reduced grip strength, ulnar neuropathy secondary to nerve compression in Guyon’s canal) frequently requires excision of the nonunited hook fragment, a simple and rapid solution for athletes but which may produce ulnar nerve lesions [18,68,70]. Complications of hook of hamate fractures also include ulnar artery thrombosis (hypothenar hammer syndrome) and flexor digitorum profundus tendon ruptures. Accordingly, prompt diagnosis and treatment are essential [5,67]. In golfers, an osteosynthesis with cannulated screw can be discussed towards the simple excision usually proposed for the rapid return to play [18].

**Figure 8:**
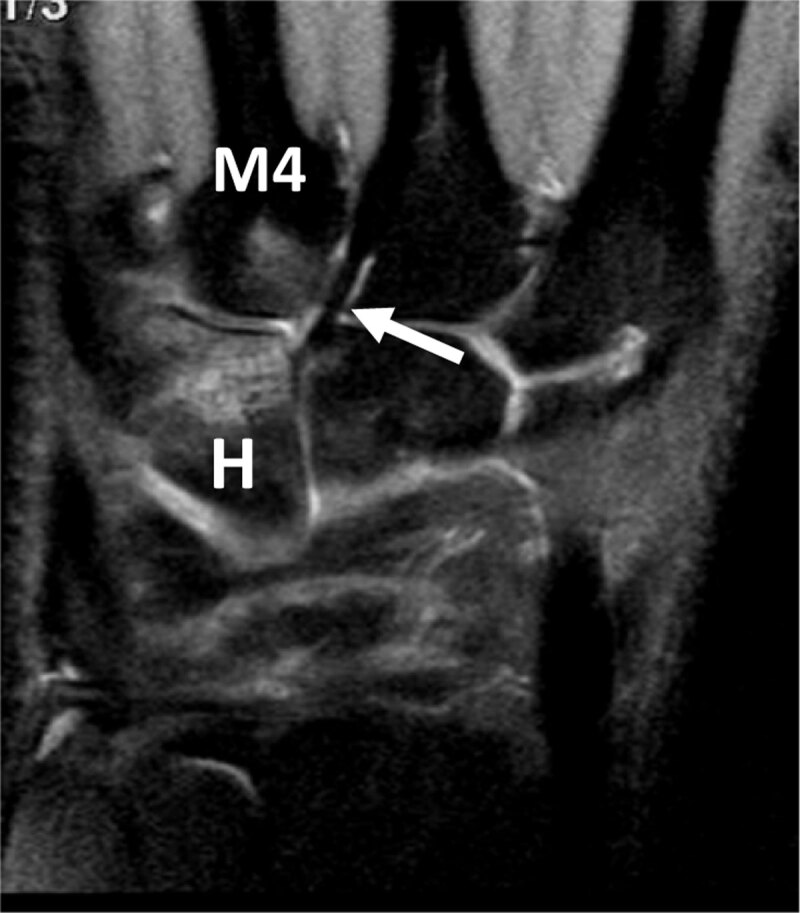
**Hamate BME and associated BME of the fourth metacarpal base in an overused pianist hand.** Coronal proton-density fat-saturated MR image depicts high signal bone marrow infiltration of the distal half of the hamatum (H) and proximal base of the fourth metacarpal (M4) nearby the capito-third metacarpal ligament (arrow) which is strong in this professional piano player.

Hamatum fracture is often associated with **carpometacarpal dislocation**, especially in motor bikers. The swollen hand being difficult to examine, this should encourage the specialist to focus on the carpometacarpal joint alignment. Plain CT is the imaging modality of choice to document hamate collapse. A rapid reduction is maintained by simple carpometacarpal and intermetacarpal pinning. Arthroscopy can provide diagnostic or therapeutic assistance [18] (***Figure 8***).

In conclusion, because of the frequently undetected wrist fractures at the emergency department, based on clinical examination and conventional radiographs, the sports physician or orthopedic surgeon and the radiologist could in specific cases, complete the diagnosis by an urgent or semi-urgent MRI examination of the wrist to detect occult fractures and/or ligament injuries. Apart from complications that will result from underdiagnosed fractures such as nonunion, SNAC, and osteonecrosis, the purpose is also not to miss the severe ligament injuries and to prevent development of carpal instability or complications as a SLAC wrist. Moreover, in acute and subacute setting (until approximately six weeks after trauma), torn ligaments might still be surgically repaired before extensive infiltration by scar tissue. After this period, surgical treatment is more invasive (arthrodesis) resulting in functional impairment.
